# Genotype-by-Environment Interaction Analysis of Metabolites in Pearl Millet Genotypes with High Concentrations of Slowly Digestible and Resistant Starch in Their Grains

**DOI:** 10.3390/cells11193109

**Published:** 2022-10-02

**Authors:** Chandra Bhan Yadav, Prakash I. Gangashetty, Manfred Beckmann, Luis A. J. Mur, Rattan S. Yadav

**Affiliations:** 1 Institute of Biological Environmental and Rural Sciences (IBERS), Aberystwyth University, Plas Gogerddan, Aberystwyth SY23 3EB, UK; 2 International Crops Research Institute for the Semi-Arid Tropics, Patancheru, Hyderabad 502324, India

**Keywords:** pearl millet, slowly digestible starch, resistant starch, nutritional traits, genotype by environment interaction, grain metabolites

## Abstract

Genotype × environment interactions (GEIs) should play an important role in the selection of suitable germplasm in breeding programmes. We here assessed GEI effects on pearl millet (*Pennisetum glaucum* L.) genotypes, selected to possess a high concentration of slowly digestible starch (SDS) and resistant starch (RS) in their grains. Entries were grown in a randomized complete block design with three replications at locations in Bawku-Ghana, Sadore-Niger, Bamako-Mali, Konni-Nigeria, and Gampella-Burkina Faso across West Africa. Harvested grains from these locations were metabolomically profiled using flow injection ionization-high-resolution mass spectrometry (FIE-HRMS). A total of 3144 mass features (*m/z*) (1560 negative ion mode and 1584 positive ion mode) were detected, of which, 475 *m/z* were linked to metabolites be involved in starch, antioxidant and lipid biosynthesis, and vitamin metabolism. Combined ANOVA revealed that the GEI was significantly evident for 54 health-benefiting metabolites, many associated with sugar, especially galactose, metabolism. Additive main effects and multiplicative interaction (AMMI) analysis examined genotype variation and GEI effects, which, when combined with principal component analysis (PCA), found that *m/z* 171.14864 (positive ionisation, propenyl heptanoate) accounted for 89% of the GEI variation along PC1. The AMMI-based stability parameter (ASTAB), modified AMMI stability value (MASV), and modified AMMI stability index (MASI) were then applied to identify stable and high-performing genotypes for all the health-benefiting metabolites. Similarly, the best-linear-unbiased-prediction (BLUP)-based stability estimation was also performed using the harmonic mean of genotypic values (HMGV), relative performance of genotypic values (RPGV), and harmonic mean of relative performance of genotypic values (HMRPGV), to identify genotype rankings across multiple environments. The multi-trait stability index (MTSI) was calculated and found that the genotypes G1 (ICMH-177111) and G24 (ICMX-207137) were the most stable and were the best mean performers across 52 health-benefiting metabolic traits. These findings demonstrate the potential of G × E assessments on the delivery of health-benefiting metabolite-rich grains in future varieties and hybrids of pearl millet.

## 1. Introduction

Pearl millet (*Pennisetum glaucum* L.) is an important staple cereal crop that is grown on more than 26 million hectares to produce more than 29 tons of grains worldwide. It is mostly grown in the arid and semi-arid tropical regions of Africa (17 million ha) and Asia (10 million ha), where drought, heat, and low-fertility soils can lead to losses in crop production [1,2,3]. Pearl millet is a climate-resilient and nutrient-rich cereal grain possessing essential nutrients such as starch, antioxidants, folate, and essential amino acids, which can enrich the diets of the populations living in emerging economies [4,5,6]. Pearl millet also has non-glutinous and non-acid forming properties and is easy to digest. Moreover, pearl millet grains are rich in nutritionally important minerals such as iron, calcium, zinc, magnesium, phosphorous, and potassium. Pearl millet grains are also a good source of dietary fibre and several vitamins (β-carotene, niacin, vitamin B6, and folic acid). Millets are also rich in polyphenols, tannins, and phytosterols and are a good source of antioxidants.

Recently, pearl millet entries possessing a high concentration of slowly digestible starch (SDS), resistant starch (RS), antioxidants, and vitamins were identified by Yadav et al. [7] in the world collection of pearl millet germplasm set known as the Pearl Millet Inbred Germplasm Association Panel (PMiGAP). The current study was planned to gain an understanding of the metabolites available in these genotypes and how their concentrations are affected by changes in environmental conditions. Multivariate statistical analysis of metabolites can provide a comprehensive overview of the relationships between metabolite components in pearl millet when grown under different environmental conditions. Crucially, such a screening strategy could be used to identify superior and stable pearl millet genotypes with enhanced nutritional components across environments. 

In the present study, we investigated metabolite diversity in eleven pearl millet hybrids known to possess high concentrations of SDS and RS in their grains. The study focused on comparative analysis of health-benefiting metabolites in the harvested grains of these entries after growing them at five different environmental locations across West Africa, but under unified agronomical practices. Hybrids were chosen for their high RS and SDS properties known to contribute to the low glycaemic index (GI) of pearl millet. 

## 2. Materials and Methods

### 2.1. Experimental Material, Field Trials, and Data Collection

Eleven pearl millet genotypes were used in this study, viz. ICMX-207076, ICMX-207094, ICMX-207136, ICMX-207137, ICMX-207171, ICMX-207181, ICMX-207183, ICMX-207190, ICMH-177111, ICMA177002, and ICMA177003 (Supplementary Table S1). The field trials were conducted with these genotypes at five locations, Bawku-Ghana, Sandore-Niger, Bamako-Mali, Kano-Nigeria, and Gampella-Burkina Faso in West Africa. Geographic co-ordinates (latitude, longitude, and altitude), type of soil, and weather data during the cropping season (total rainfall, minimum and maximum temperature) for each location are given in Supplementary Table S2. The experiment used 8 hybrids along with the parental genotypes that were sown under field conditions in a randomized complete block design (RCBD) with three replications during the 2018 rainy season. Each genotype was sown in 2-row plots of 3 m in length, with a 0.75 m distance between the rows and 0.20 m in-between plants. Test plots were thinned at 12 days after seedling emergence (DAE) to one plant per hill with a plant population of 30 per plot. A basal dose of fertilizer, 100 kg/ha (NPK), was applied to the fields at the land preparation stage. Micro-dosing of the crop with urea at 2 g per hill was carried out at 30 DAE. 

### 2.2. Metabolite Fingerprinting

Metabolite extraction was performed from frozen milled grain samples of eleven accessions of pearl millet. Three biological replicates were used to minimize any measurement errors. Pearl millet grains in an amount of 50 mg ± 1 mg were flash frozen in liquid N_2_ and homogenized using a ball mill. The samples were placed on ice, and 1 mL of extraction solution (chloro-form/methanol/water, 1:2.5:1, *v/v/v*) was added followed by incubation at 4 °C for 15 min with constant stirring. The aqueous supernatants were collected after centrifugation at 21,000× *g* for 5 min at 4 °C and returned to the ice. Then, 100 µL of the extracted samples was analysed using flow infusion electrospray ionization high-resolution mass spectrometry (FIE-HRMS). The four independent replicates were considered for each sample, and metabolite fingerprinting was performed by FIE-HRMS using a Q Exactive Plus Hybrid Quadrupole Orbitrap Mass Analyser with an Accela UHPLC system (Thermo Fisher Scientific, Bremen, Germany). The sample was injected into the capillary column in a randomized order, and the *m/z* (mass ion) values were recorded in both positive and negative ionization modes as described by Skalska et al. [8] and Yadav et al. [9]. The peak area of each metabolite was analysed using the MetaboWorkflows R package version 0.9.5 (https://jasenfinch.github.io/metaboWorkflows, accessed on 18–25 March 2022) for spectral processing, data pre-treatment, and quality control. Statistical analyses for metabolites’ diversity were performed using MetaboAnalystR (http://www.metaboanalyst.ca, accessed on 18–25 March 2022), as described in Yadav et al. [9]. Briefly, metabolite *m/z* values were normalized as a percentage of total ion count, and values were transformed to log_10_ and Pareto scaling. Significant metabolites were identified by cross-validated p-values (adjusted using Bonferroni correction to reduce false positives), based on one-way analysis of variance (ANOVA), setting the significance at *p* < 0.05. The functional pathway of each untargeted metabolite was identified by applying the functional analysis module of MetaboAnalyst 5.0 using the reference library: Oryza sativa. The selection of metabolites contributing to health-benefiting traits was as described by Yadav et al. [9]. 

### 2.3. Statistical Analysis for Phenotypic Variance and Heritability

The phenotypic variance was analysed using a mixed linear model implemented in the LME4 R-package that uses REML to estimate the variance components. ANOVAs were performed on replicated data observed in different environments (five locations). The analysis was performed for individual environment, as well as combined across environments, in a random complete block design (RCBD). In combined analysis across environments, the genotype interactions with the environments were considered as random effects [10,11,12].

The broad-sense heritability was calculated for each health-benefiting metabolite in an individual environment as: $H^{2}=\frac{{\sigma^{2}}_{g}}{{\sigma^{2}}_{g}+{\sigma^{2}}_{\varepsilon }/\eta \mathrm{Rep}}$, where σ^2^_g_ and σ^2^_ε_ are the genotype and error variance components, respectively, and ηRep denotes for replicates. The combined heritability analyses were calculated as: $H^{2}=\frac{{\sigma^{2}}_{g}}{{\sigma^{2}}_{g}+{\sigma^{2}}_{\mathrm{ge}}/\eta \mathrm{Loc}+{\sigma^{2}}_{\varepsilon }/(\eta \mathrm{Loc}\;\times \;\eta \mathrm{Rep})}$ in multiple environment factors. Here, σ^2^_ge_ is the GEI variance component and ηLoc is the number of environments [13,14]. 

The REML model was applied for observation of the best linear unbiased predictors (BLUPs) for each genotype, thus influencing the effect of the adjacent rows [15].

### 2.4. Additive Main Effects and Multiplicative Interaction Model 

The additive main effects and multiplicative interaction (AMMI) model was implemented in the metan r package [15], which is the most advanced model for additive main effects and multiplicative analysis. This involves fitting an additive model (ANOVA) for general means, G and E means, and a multiplicative model (i.e., PCA) for the residuals of an additive model or a genotype, environment, and their interactions [16]. Patterson and Williams [17] implemented a model to observe the response variable for the *i*th genotype in the *j*th environment (*yij*) calculated as: $yij=\mu +\alpha i+\tau j+\sum_{k=1}^{p}\lambda_{k\;}a_{ik}\;t_{jk}+\rho_{ij}+\varepsilon_{ij}$, where λ_k_ is the singular value for the *k*th interaction principal component axis (IPCA); aik is the ith element of the *k*th eigenvector; *t_jk_* is the *j*th element of the kth eigenvector. A residual *ρij* remains, if not all *p* IPCA are used, where *p* ≤ min (g − 1; e − 1). An additive main effects model along with a multiplicative model (i.e., PCA) explained the genotype environment interaction effect of each genotype and partitioning of their interaction effects in response to individual environments.

### 2.5. Genotype plus Genotype-by-Environment Model on Multi-Environment Factor 

GGE modelling strategies are based on multi-environment trial data that include GEI variation as a combination of the genotypic main effect and the GEI as a sum of the multiplicative response (i.e., PCA). GGE biplots provide graphical depictions that allow the evaluation of genotype performance in different environments. This allows the association between specific environments and between genotypes to be explained. GGE biplots were generated using Genstat (18th edition) [18] by selecting the convex hull around genotype scores option to mark the convex hull around the genotype scores, the sectors to divide the biplot into segments, the mega environment to draw an ellipse round those environments that share the same sector, and linking the environment based on its scores with the origin.

### 2.6. Estimation of AMMI- and BLUP-Based Stability

Several AMMI-based stability methods were implemented in the Metan r package by [15]. The AMMI-based stability parameter $\left(\mathrm{ASTAB}=\sum_{n=0}^{N^{\prime }}\lambda_{n\;}\gamma_{in}^{2}\right)$ by Rao and Prabhakaran [19], Annicchiarico’s D parameter $D_{a}=\sqrt{\sum_{n=0}^{N^{\prime }}{\left(\lambda_{n\;}\gamma_{in}^{\;\;\;\;}\right)}^{2}}$ by Annicchiarico [20], Zhang’s D parameter ($D_{z}=\sqrt{\sum_{n=0}^{N^{\prime }}\lambda_{in}^{2}}$) by Zhang et al. [21], the AMMI stability index ($\mathrm{ASI}=\sqrt{[{\mathrm{PC}}_{1}^{2}\times \theta_{1}^{2}]+[{\mathrm{PC}}_{2}^{2}\times \theta_{2}^{2}]}$) by Jambhulkar et al. [22], the weighted average of absolute scores $\left(WAAS_{i}=\sum_{k=1}^{N}|IPCA_{ik\;}\times \theta_{k}/\sum_{k=1}^{p}\theta_{k}\right)$ by Olivoto et al. [23], the sums of the averages of the squared eigenvector values $\left(EV=\sum_{n=1}^{N^{\prime }}\frac{\gamma_{in}^{2}}{N^{\prime }}\right)$ by Zobel [24], the stability measure based on the fit AMMI model $\left(FA=\sum_{n=1}^{N^{\prime }}\lambda_{n}^{2}\gamma_{n}^{2}\right)$ by Raju [25], the modified AMMI stability index $(\mathrm{MASI}=\sqrt{\sum_{n=1}^{N^{\prime }}(PC_{n}^{2}\times \theta_{n}^{2})}$ by Ajay et al. [26], and Spearman’s rank correlations among all 13 stability values were derived.

The best-linear-unbiased-prediction (BLUP) based stability estimation method was implemented in the Metan r package for the selection of stable genotypes with the best performance in a mixed effects model structure. Colombari et al. [27] (2013) demonstrated the use of three BLUP-based indexes for the selection of genotypes for performance and stability. The first methods involve the prediction of the harmonic mean of genotypic values $\left(HMGV_{i}=\frac{E}{\sum_{j=1}^{E}\;{\frac{1}{Gv}}_{ij}}\;\right),$ the second RPGV index $\left(RPGV_{i}=\frac{1}{E}\sum_{j=1}^{E}Gv_{ij}/\mu_{j}\;\right),$ and the HMRPGV index $\left(HMRPGV_{i}=\frac{E}{\sum_{j=1}^{E}\frac{1}{Gv_{ij}/\mu_{j}}}\right)$. *E* represents the number of environments; *Gv_ij_* is the genotypic value (BLUP) for the *i*th genotype in the *j*th environment. 

### 2.7. Multi-Trait Stability Analysis

The multi-trait stability index (MTSI) by Olivoto and Lúcio [15] was implemented in the Metan r package to predict the stability and mean performance across all the traits together. The MTSI was estimated using the WAASBY index based on the superiority index based on the mixed effects model $WAASBY_{i}=\frac{\left(rG_{i}\times \theta_{Y}\right)+\left(rW_{i}\times \theta_{S}\right)}{\theta_{Y}+\theta_{S}}$ described by Olivoto et al. [23].

## 3. Results

### 3.1. Metabolite Profiling 

Eleven pearl millet genotypes were selected based on high RS and SDS traits by Yadav et al. [28]. The cultivars were grown in five locations in West Africa (Bawku-Ghana, Sadore-Niger, Bamako-Mali, Konni-Nigeria, Gampella-Burkina Faso), and metabolite content was evaluated using flow infusion electrospray high-resolution mass spectrometry (FIE-HRMS). A total of 3144 mass features (*m/z*) (1560 negative ion mode and 1584 positive ion mode) were assessed based on the presence in at least three replicates of each genotype of pearl millet. Metabolites were tentatively identified, and pathway enrichment factor analysis using the mummichog module of Metaboanalyst (https://shuzhao-li.github.io/mummichog.org, accessed on 18–25 March 2022) linked 475 mass features to metabolites linked with human health benefits (Supplementary Table S3). 

**Figure 1:**
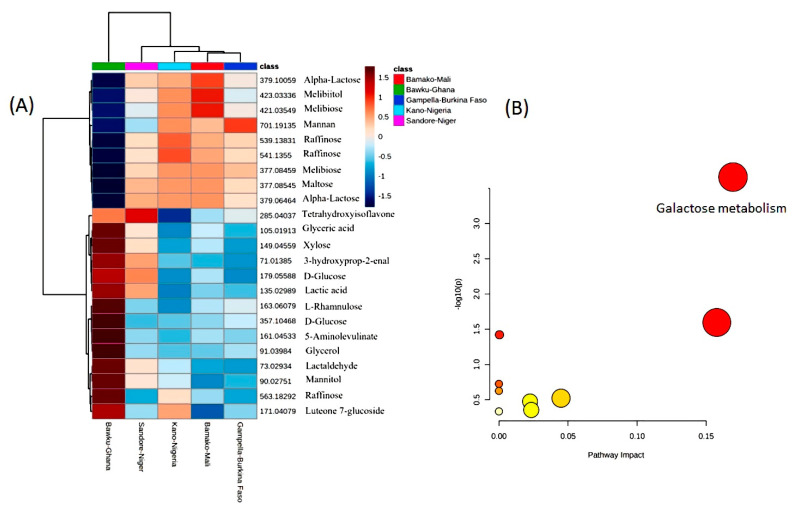
(**A**) Heatmap visualization of the mean performance of the 25 health-benefiting metabolites identified across five locations in pearl millet lines. Each value represents the normalized (median-centred and log_10_-transformed) mean of three biological replicates, with red and blue colours denoting relatively high and low intensities (Loc1-Bawku-Ghana, Loc2-Sadore-Niger, Loc3-Bamako-Mali, Loc4-Konni-Nigeria, Loc5-Gampella-Burkina Faso). (**B**) Biochemical pathway enrichment assessments of the metabolites shown in (**A**). The circle sizes and deeper colours indicate increasingly significantly and biochemical important pathways.

Out of the 475 metabolites, 97 metabolites (65 negative ion and 32 positive ion modes) were targeted as being involved in “starch and sucrose”, “galactose”, and “fructose and mannose” metabolism through pathway enrichment analysis. Similarly, 115 mass features (63 negative ions and 52 positive ion mode) were associated with antioxidant biosynthesis pathways (such as anthocyanins, carotenoids, glutathione, flavonoids, flavones, and flavonols) and 131 metabolites (73 negative ions and 58 positive ion mode). Other important pathways were linked to vitamin metabolism (ascorbate, biotin, riboflavin (vitamin B2)), pyridoxine (vitamin B6), folate (vitamin B9), lipid metabolism (“fatty acid degradation”, “fatty acid biosynthesis”, biosynthesis of unsaturated fatty acids), nitrogen metabolism, inositol phosphate, and zeatin biosynthesis (Supplementary Table S3). Two-way ANOVA allowed the ranking of 25 metabolites that showed the most variation across the locations (Figure 1A). Pathway enrichment and impact assessments indicated the importance of galactose metabolism (*p* = 2.1874 × 10^−4^, FDR = 0.02078) (Figure 1B). These preliminary assessments indicated the existence of G × E effects that required further assessment. 

### 3.2. ANOVA for Phenotypic Variance

The 475 metabolites were subjected to a combined ANOVA analysis for significance at *p* < 0.05 for both factors (genotypes (G) and environments (E)) and GEI (genotype and environment interaction) to define multi-factor responses in these genotypes in the five different environmental conditions. Combined analyses of variance across the environments suggested that genotypic, environmental, and interactions were significant for 52 health-benefiting metabolites (Supplementary Table S4). Of these, 13 metabolites (10 negative ions and 3 positive ion mode) that were involved in starch and sucrose metabolism showed significant (G), (E), and (GEI) differences. Similarly, 13 mass features (7 negative ions and 6 positive ion mode) associated with antioxidant biosynthesis pathways, 21 metabolites (10 negative ions and 11 positive ion mode) with vitamin metabolism, and 7 metabolites (3 negative ions and 4 positive ion mode) linked with lipid biosynthesis showed that genotype, environment, and their interactions were highly significant at *p* < 0.001. 

The high broad-sense heritability observed for the 52 metabolites indicated that the genotypic differences detected are primarily due to genetic effects. The current analysis showed that *m/z* 164.04323 (negative ionization, tentatively identified as the vitamin 6 metabolite, 4-pyridoxolactone) was strongly heritable (>0.88) across five environments, whereas broad-sense heritability for *m/z* 164.04323 was higher (>0.87) in an individual environment (Sadore, Niger). However, when assessed on the basis of a pooled environment, a partitioning of the GEI component lowered the heritability for both traits across environments (Supplementary Figure S1). 

### 3.3. Additive Main Effects and Multiplicative Interaction Analysis 

At the same time as the ANOVA based on an additive model, which illustrates the effect of the source of variation, the AMMI model emphasizes the pattern of genotypes (G) and/or environments (E) and their interactions. AMMI-based statistical analysis for 54 health-benefiting metabolites showed significant variation within the eleven genotypes across the five environments (Supplementary Table S5). AMMI-based combined ANOVA revealed that environment, genotype, and GEI showed significant variation at 0.1% (*p* < 0.001) for 40 metabolites, whereas 9 metabolites showed non-significant genotype and environment interactions (Supplementary Table S5). The G × E, as well as GEI variations were significant at *p* < 0.001 for most of the metabolites. AMMI-based analysis revealed that GEI had major contributions from the first principal components (PCs), which ranged from 39.9 to 91.2% for health-benefiting metabolites. One *m/z* (positive ionisation, 171.14864, tentatively identified as the fatty acid ester, propenyl heptanoate) showed the maximum variance with the first PC (which explained 91.2% of the total GEI), while the second, third, and fourth PCs contributed 7.2, 1.0, and 0.7%, respectively (Supplementary Table S5). Genotype contributed 18.38% to the total variation of *m/z* p171.14864, whereas the environment contributed major variations (33.07%), and GEI contributed 4.84%. Compared to other mass features, PC1 for *m/z* 380.1561 (negative ionisation; tentatively identified as cis-zeatin-9-N-glucoside) explained the least variance at only 39.9% of the total GEI, while the second, third, and fourth PCs contributed 26.9, 18.4, and 14.9%, respectively. Genotypic variance explained 13.70% for *m/z* 380.1561; however, the environment and GEI contributed about 15.78 and 2.34%, respectively.

**Figure 2:**
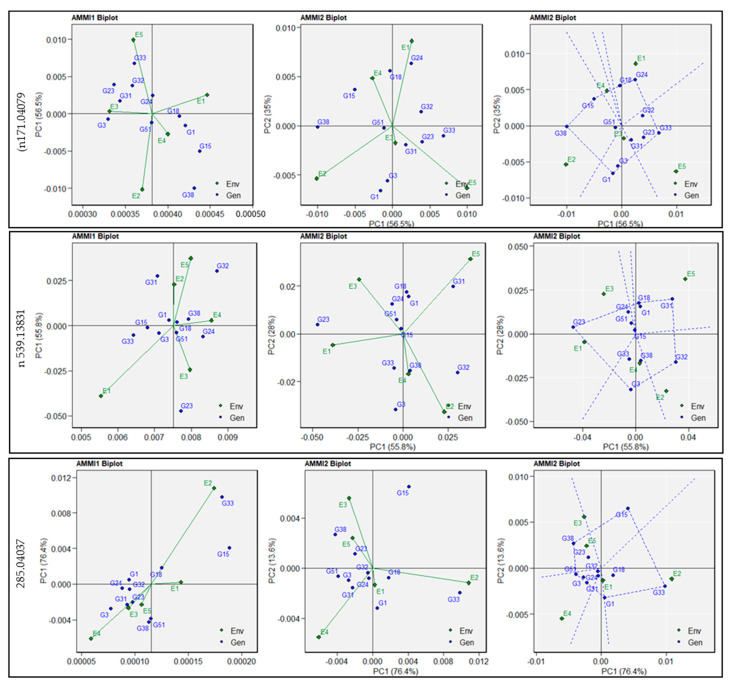
Additive main effects and multiplicative interaction (AMMI) for metabolites linked to antioxidant (n171.04079), starch (n539.13831), and vitamin metabolism (n285.04037) in 11 pearl millet genotypes evaluated in five environments. AMMI-based biplot generated using AMMI1 biplot (trait vs. Principal Component 1 (PC1)), AMMI2 biplot (PC1 vs. PC2), and AMMI2 biplot (PC1 vs. PC2) with the polygon option.

AMMI1 biplots were generated to explain the interactive correlation between genotypes and environments for the 54 metabolites (Supplementary Figure S2a–e). The analyses showed distinct patterns amongst genotypes interacting across the environments for *m/z* 171.04079 (negative ionisation), tentatively identified as luteone 7-glucoside, involved in the antioxidant pathway, and *m/z* 539.13831 (negative ionisation), tentatively identified as raffinose and involved in starch metabolism. Similar environmental effects for *m/z* 285.04037 (negative ionisation, tentatively identified as 7, 8, 3’, 4’-tetrahydroxyisoflavone) were observed except with E2 (Sadore-Niger), which had a distinct effect on genotype adaptation (Figure 2). For *m/z* 285.04037, E2 (Sadore-Niger) and E4 (Konni-Nigeria) were the furthest from the biplot origin, which indicated strong interaction influences for genotype adaption. E1 (Bawku-Ghana), E3 (Bamako-Mali), and E5 (Gampella-Burkina Faso) were closest to the biplot origin point and had shorter vectors, indicating weak interaction with genotype adaptation. The AMMI2 biplot described the performance of the entries and their adaptation to a specific environmental condition (Figure 2). 

The AMMI2 biplot for the antioxidant metabolism metabolite (*m/z* 171.04079) showed that the genotype G24 (ICMX-207137) showed the best performance in the E1 (Bawku-Ghana) environment. Genotype G33 (ICMX-207183) had the highest levels, as this showed good adaptation in E5 (Gampella-Burkina Faso). In contrast, genotypes, namely G15 (ICMX-207076), G38 (ICMX-207190), and G1 (ICMH-177111), had higher *m/z* 285.04037 content, but negative adaptability across the environments such as E2 (Sadore-Niger), E3 (Bamako-Mali), and E4 (Konni-Nigeria). The AMMI2 biplot for the metabolite involved in starch metabolism (*m/z* 539.13831, raffinose) and genotypes such as G1 (ICMH-177111), G18 (ICMX-207094), G31 (ICMX-207171), and G32 (ICMX-207183) exhibited good performance in E2 (Sadore-Niger) and E5 (Gampella-Burkina Faso). However, with genotypes, G23 (ICMX-207136) and G3 (ICMX-207207) had higher levels of *m/z* 539.13831, but had negative adaptabilities across certain environments (E1-Bawku-Ghana, E3-Bamako-Mali, E4-Konni-Nigeria). Similarly, for *m/z* 285.04037, genotypes such as G33 (ICMX-207183) had the highest degree of performance with high-ranking adaptability in E2, but G38 (ICMX-207190) was associated with negative adaptability in E3 (Bamako-Mali) and E5 (Gampella-Burkina Faso).

### 3.4. Genotype plus Genotype-by-Environment Biplots

**Figure 3:**
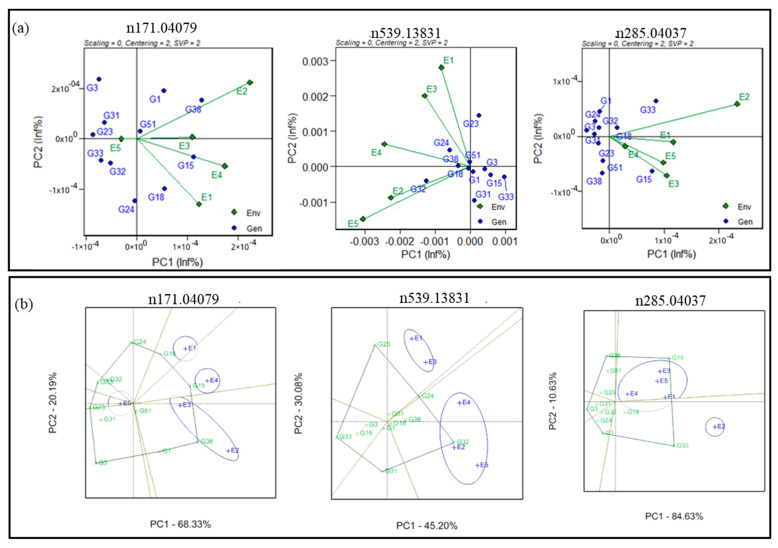
Genotype-by-genotype × environment (GGE) biplot (**a**) to identify the favourable environment for antioxidant-(n171.04079), starch-(n539.13831), and vitamin-(n285.04037) linked metabolites and (**b**) polygon view biplot for identification of stable genotypes for antioxidant-(n171.04079), starch-(n539.13831), and vitamin-(n285.04037) associated metabolites across the testing environments.

Genotype-by-environment (GGE) biplot analysis of multiple traits was used to compare multiple genotypes in multiple environments (five environments) for the 54 health-benefiting metabolites (Supplementary Figures S2 and S3). GGE-based biplots were generated to connect environment scores with the origin and found favourable environmental effects for *m/z* 171.04079 and *m/z* 285.04037. However, contrastingly, unfavourable environmental effects were observed for the starch-metabolism-related (*m/z* 539.13831) metabolite (Figure 3a). The GGE biplots also indicated that E2 (Sadore-Niger) and E3 (Bamako-Mali) showed a positive correlation for *m/z* 285.04037 metabolite levels, but a negative correlation with E1 (Bawku-Ghana) and E2 (Sadore-Niger).

The GGE biplots also indicated the best-performing genotypes in each of those environments by highlighting the convex hull around genotype scores, sectors, and mega environments that share the same sector (Supplementary Figure S4a,b). For example, the first two interaction principal component axes (IPC) accounted for more than 88.51% of the G + GE sum of squares for *m/z* 285.04037. The biplot showed four mega environments with E3 (Bamako-Mali) and E5 (Gampella-Burkina Faso) sharing a common mega environment and having a similar effect on *m/z* n285.04037. Similarly, the first two IPCs explained 75.28% (genotype–environment interaction) for the starch metabolism (*m/z* 539.13831) and 95.27% of the GEI for *m/z* 285.04037. The convex hull biplot exhibited two mega environments for *m/z* 539.13831 in which E1 (Bawku-Ghana) and E3 (Bamako-Mali) share a common mega environment and E2 (Sadore-Niger), E4 (Konni-Nigeria), and E5 (Gampella-Burkina Faso) are assigned together to have effects on the starch metabolism (*m/z* 539.13831) metabolite (Figure 3b). The convex hull biplot exhibited two mega environments for *m/z* 285.04037 in which E1 (Bawku-Ghana), E3 (Bamako-Mali), E4 (Konni-Nigeria), and E3 (Bamako-Mali) share a common mega environment and were different in E2 (Sadore-Niger). 

### 3.5. Estimation of AMMI-Based Stability Indices

The AMMI-based stability was performed to rank the genotypes for their performance across the different environments. Various AMMI-based statistical models and parameters were applied, namely ASI, ASV, ASTAB, AVAMGE, DA, DZ, EV, FA, MASI, MASV, SIPC, and Za (Supplementary Table S6). The genotype G51 (ICMX-207192) was the most stable, and G31 (ICMX-207171) ranked second, when using each AMMI-based stability assessment except ASV and MASV for *m/z* 285.04037. For the starch-metabolism-related compound (*m/z* 539.13831), G15 (ICMX-207076) was ranked 1st and G51 (ICMX-207192) 2nd for their performance across the various environments. Similarly, the genotype G32 (ICMX-207181) ranked 1st and G51 (ICMX-207192) 2nd and showed the highest stability for the vitamin-metabolism-related metabolite *m/z* 593.15131 (Supplementary Table S6). 

### 3.6. Estimation of Best-Linear-Unbiased-Prediction-Based Stability Indices

The BLUP-based analysis was performed with BLUP-based statistical models to estimate highly stable genotypes across multiple environmental conditions (Supplementary Figure S5). The ranking of the genotypes was as follows: G15 (ICMX-207076) 1st, G32 (ICMX-207181) 2nd, G1 (ICMH-177111) 3rd, and G18 (ICMX-207094) 4th as highly stable genotypes for *m/z* 171.04079 according to HMGV, RPGV, and HMRPGV, but not the WASSB method. Similarly, genotypes ranked as 1st G32 (ICMX-207181), 2nd G31 (ICMX-207171), 3rd G38 (ICMX-207190), and 4th G23 (ICMX-207136) were identified as highly stable genotypes for starch metabolism *m/z* 539.13831 by HMGV, RPGV, and HMRPGV, but not the WAASB method. For the vitamin-associated metabolite (*m/z* 593.15131), G32 (ICMX-207181) ranked 1st, G31 (ICMX-207171) 2nd, G38 (ICMX-207171) 3rd, and G23 (ICMX-207136) 4th using HMGV, RPGV, and HMRPGV stability predictions (Supplementary Table S7). 

### 3.7. Best-Performing and Highly Stable Entries

**Figure 4:**
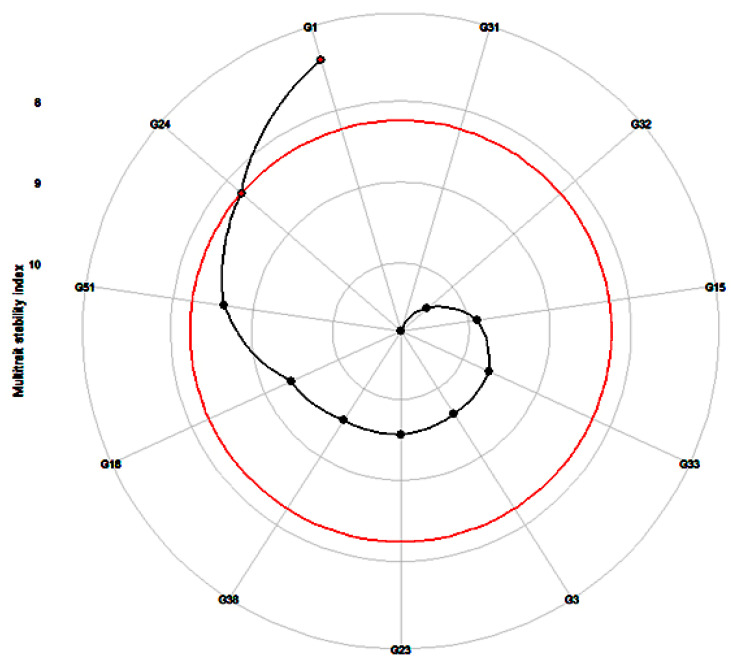
Prediction of highly stable and best-performing genotypes (G1 = ICMH-177111 and G24 = ICMX-207137) across the 52 metabolites in pearl millet lines tested in five locations. (G3 = ICMX-207207, G15 = ICMX-207076, G18 = ICMX-207094, G23 = ICMX-207136, G31 = ICMX-207171, G32 = ICMX-207181, G33 = ICMX-207183, G38 = ICMX-207190, G51 = ICMX-207192).

The MTSI was performed by the WAASBY index for genotype stability and mean performance across the 54 health-benefiting metabolites. This suggested that the genotypes G1 (ICMH-177111) and G24 (ICMX-207137) had the best stability and performance in the five environments. The stability index score was lowest for G32 (ICMX-207181), showing poor stability and mean performance in multiple environmental conditions (Figure 4).

## 4. Discussion

Millets, particularly pearl millet, are important due to their critical role in human nutrition. Substantial progress in nutritionally rich crop production has been achieved through the exploitation of classical genetics and the selection of crop varieties with improved health-benefiting traits. However, the increasing worldwide population and adverse climatic conditions have driven the need to protect and augment health-benefiting traits in micronutrient-rich grains such as pearl millet. Numerous studies have suggested that capturing naturally occurring genetic variation could be important for nutritional values, especially when considering genetic interactions in improving desired attributes [29]. Thus, this study was conducted with the aim to identify the best-performing and most-stable genotypes in multiple environments for various health-benefiting metabolites in pearl millet. 

Previously, we used metabolomic-based quantifications of health-benefiting nutritional metabolites as phenotypic traits in conjunction with 76K SNP variants to conduct metabolic genome-wide association analyses [9]. This found significant SNPs associated with health-benefiting nutritional metabolites at the −log *p*-value ≤ 4.0. The study revealed 738 probable candidate genes, which had significant roles in starch, antioxidant, vitamin, and lipid metabolism. These genes encoded starch branching, α-amylase, β-amylase, vitamin-K reductase, UDP-glucuronosyl, UDP-glucosyl transferase (UGTs), L-ascorbate oxidase, and isoflavone 2’-monooxygenase. However, environmental impacts on the levels of the associated metabolites were not considered in that study and therefore was the aim of the current study. To address G × E and GEI effects, we quantified health-benefiting antioxidant, polyphenol, vitamin, and starch-associated, especially dietary starch, metabolites in eleven pearl millet lines at five growing locations in West Africa using FIE-HRMS profiling. 

The analysis of variance indicated the existence of homogenous error variance for all the health-benefiting metabolites in each of the five environments. Further, the combined ANOVA analysis showed that the mean sum of squares due to genotype, environment, and G × E were significant for 54 health-benefiting metabolites features involved in starch, antioxidant, lipid biosynthesis, and vitamin metabolism (Supplementary Table S2). Further, GEI effects were determined using AMMI-based models to reveal if there were substantial genotypic variations amongst the environments. Combined AMMI-based ANOVA (Table S1) revealed that all components of variations—environment (location), genotype, and GEI—were highly significant. Interestingly, the percent sum of squares linked to environment (71.42%) was much greater than genotypes (16.04%) and GEI (2.11%) for *m/z* 285.04037. This signified that the environmental factor contributed sufficient variations, which was explained by genotypes’ interaction with their respective environment. Further, comparatively less variation in the genotype was found due to other factors such as differences in rainfall and temperature, and this led to different patterns of genotypic performances. The large proportion of total variation linked to the venvironment was also reported by Adugna et al. [30], Molla et al. [31], Dagnachew et al. [32], Birhanu et al. [33], Lakew et al. [34], and Seyoum et al. [35] in finger millet. Significant GEIs were also observed by Tolessa et al. [36] in pea and Singamsetti et al. [37] in maize.

Several other statistical models, namely ASI, ASV, ASTAB, AVAMGE, DA, DZ, EV, FA, MASI, MASV, SIPC, and Za, were also applied to understand the stability of genotypes. For all the genotypes, most of the stability parameters showed consistent prediction for genotypic ranking, except ZA and DA. Based on genotypic stability score estimates, G1 (ICMH-177111), G24 (ICMX-207137), G18 (ICMX-207094), and G38 (ICMX-207190) were identified as top-ranking in terms of stability for most of the health-benefiting metabolites. Such AMMI-based stability was also used by Anuradha et al. [29] in finger millet and identified the top ten of 60 genotypes for yield parameters. Similarly, Cheloei et al. [38] used similar approaches to report stability indices in rice genotypes. 

BLUP-based stability methods such as HMGV, RPGV, and HMRPGV further represent robust statistical approaches for predicting stability [13,29,39]. These methods identify genotypic effects and allow the ranking of genotypes concerning their performance [40]. In our study, the BLUP-based models showed that the ranking of genotypes had similar trends for all the genotypes with respect to each metabolite’s genotypic stability score estimates. BLUP-based stability prediction displayed that the genotypes G15 (ICMX-207076), G33 (ICMX-207183), G38 (ICMX-207190), and G23 (ICMX-207136) has top ranks in BLUP-based stability prediction for most of the health-benefiting metabolites. Similarly, the HMGV, RPGV, and HMRPGV models were applied to predict the stable genotypes and ranking by Rosado et al. [41] in macaw palm and Jatropha by Alves et al. [42]. 

The multi-trait stability index (MTSI) was calculated to identify the best-performing genotypes across various environments for multiple traits. In this study, have found that G1 (ICMH-177111) and G24 (ICMX-207137) are the most stable and best mean performers across the 52 health-benefiting metabolite traits. Our study had similar conclusions to that of Szareski et al. [43], which also reported the stability indices to predict the favourable adaptation and stability for multiple traits in wheat genotypes.

## 5. Conclusions

By combining metabolomics tools, we extensively characterized the metabolite profiles of eleven pearl millet hybrids by growing them in five distinct locations in West Africa. Multivariate statistical models applied to the data dissected the genetic and environmental effects on the accumulation of health-benefiting metabolites in these pearl millet entries. The study identified the correlation of individual environmental diversity with the metabolite diversity. The AMMI-based models used in the study identified better-adapted hybrids for West African regions. This study for the first time characterized G × E interactions on health-benefiting metabolites’ accumulation in pearl millet entries and identified hybrids that exhibit stable preferences across locations. Such information is set to be very important for breeders interested in breeding health-benefiting traits in pearl millet varieties. 

## Data Availability

The data presented in this study are available as Supplementary Files.

## Supplementary Materials

The following Supporting Information can be downloaded at: https://www.mdpi.com/article/10.3390/cells11193109/s1, Figure S1: Heritability for 52 mass features (health-benefiting metabolites) of pearl millet lines tested at five locations; Figure S2: Additive main effects and multiplicative interaction (AMMI) for (**a**–**e**) 52 metabolites in 11 pearl millet genotypes evaluated in five environments. A. Plotted using AMMI1 biplot (trait vs. Principal Component 1 (PC1)]. B. AMMI2 biplot (PC1 vs. PC2); C. AMMI2 biplot (PC1 vs. PC2) with polygon option; Figure S3: Genotype-by-genotype × environment (GGE) biplot to identify the favourable environment for all the health-benefiting metabolites involved in antioxidant, starch, and vitamin metabolism. Metabolite identity code mentioned in Figure S2; Figure S4: Genotype-by-genotype × environment (GGE) polygon view biplot (**a**,**b**) for identification of stable genotypes for antioxidant, starch and vitamin metabolism across the testing environments. Metabolite identity code mentioned in Figure S2; Figure S5: Predicted genetic values (BLUPs) were estimated to guide the inferences based on multiple environments; Table S1: Details of the 11 different accessions, which is part of the Pearl Millet Inbred Germplasm Association Panel (PMiGAP) used in the trails at five different locations; Table S2: Geographic coordinates and climatic data of the experimental sites in Africa; Table S3: Metabolite pathway analyses to identify the health-benefiting metabolites (negative and positive ion mode) in pearl millet lines using the mummichog algorithm; Table S4: Combined analysis of variance for health-benefiting metabolites significant at *p* < 0.05 in pearl millet; Table S5: Additive-main-effects and multiplicative-interaction (AMMI)-based ANOVA analysis for health-benefiting metabolites in pearl millet genotypes in the five test environments; Table S6: AMMI-based stability estimates for ranking the pearl millet genotypes evaluated at the five test locations; Table S7: BLUP-based stability estimates for ranking the pearl millet genotypes evaluated at the five test locations.
