# Supplementary material for: Genotype-by-Environment Interaction Analysis of Metabolites in Pearl Millet Genotypes with High Concentrations of Slowly Digestible and Resistant Starch in Their Grains
Source: Cells. 2022 Oct 2;11(19):3109. doi: 10.3390/cells11193109 (PMC9563316; doi:10.3390/cells11193109)
Supplement: Supplementary file 1 [file cells-11-03109-s001.zip › Suppl_Figures.pptx]

## Slide 1
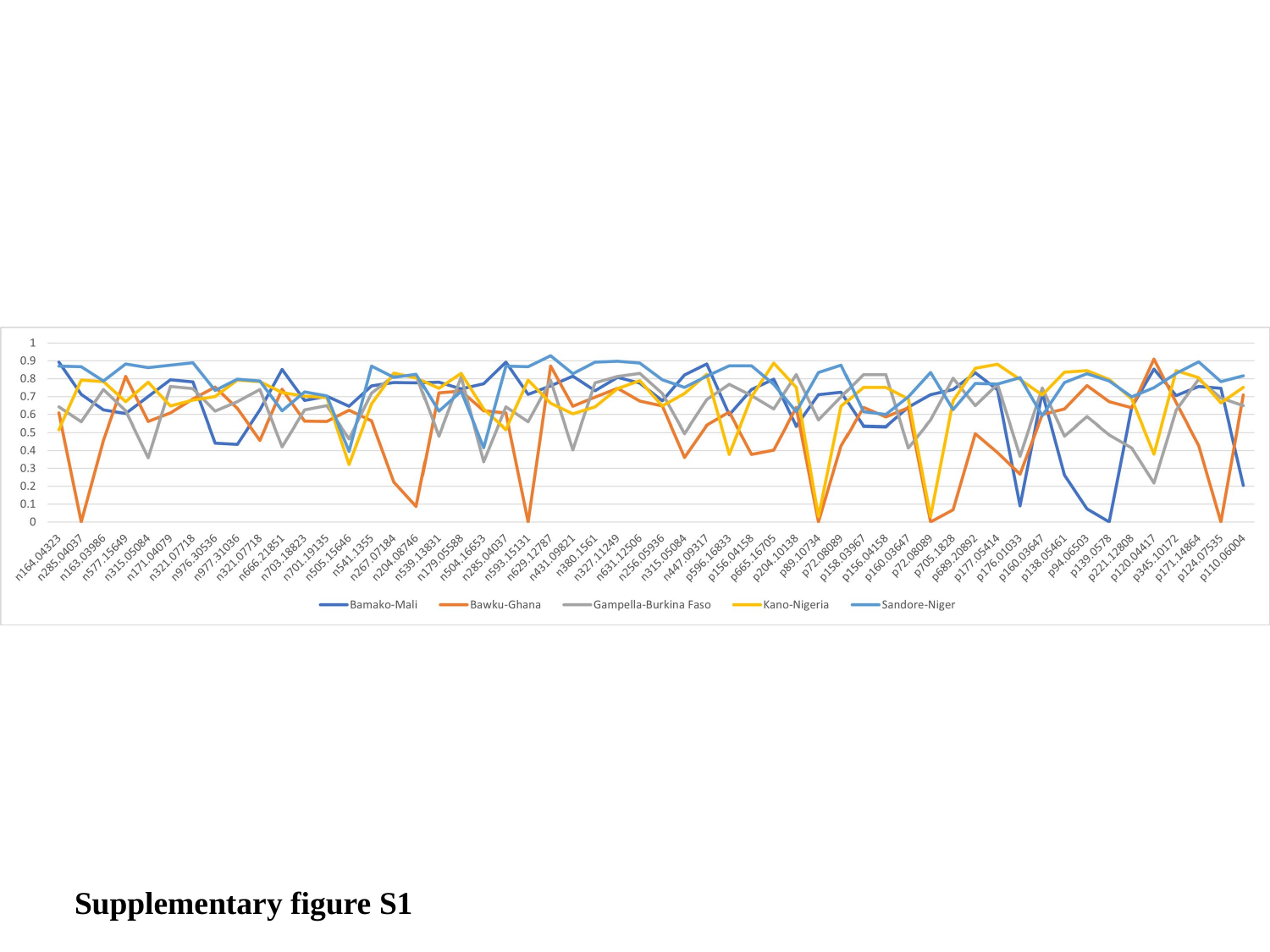

Supplementary figure S1

## Slide 2
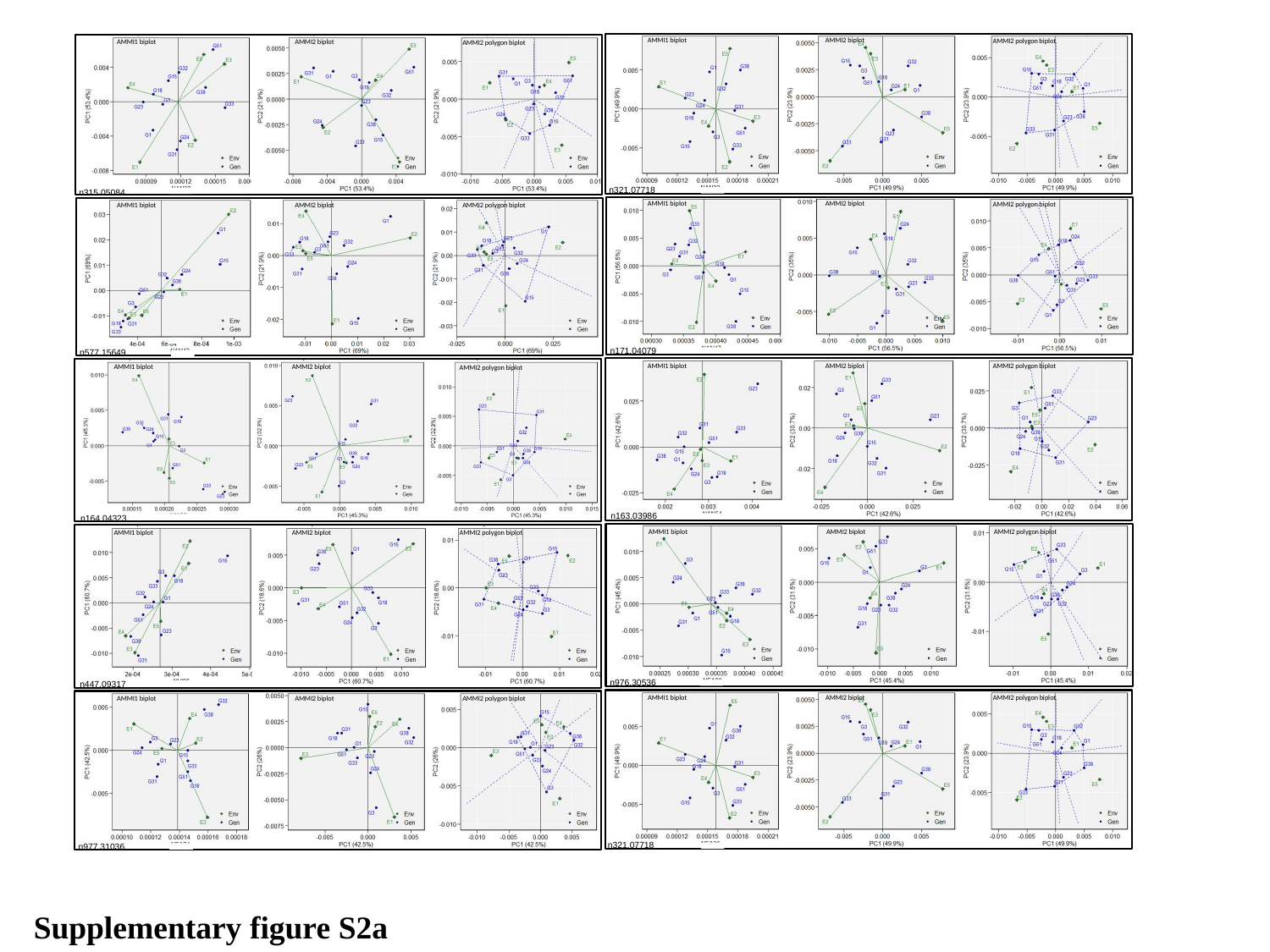

AMMI1 biplot
AMMI2 biplot
AMMI2 polygon biplot
AMMI1 biplot
AMMI2 biplot
AMMI2 polygon biplot
n321.07718
n315.05084
AMMI1 biplot
AMMI2 biplot
AMMI2 polygon biplot
AMMI1 biplot
AMMI2 biplot
AMMI2 polygon biplot
n171.04079
n577.15649
AMMI1 biplot
AMMI2 biplot
AMMI2 polygon biplot
AMMI1 biplot
AMMI2 biplot
AMMI2 polygon biplot
n163.03986
n164.04323
AMMI1 biplot
AMMI2 biplot
AMMI2 polygon biplot
AMMI1 biplot
AMMI2 biplot
AMMI2 polygon biplot
n976.30536
n447.09317
AMMI1 biplot
AMMI2 biplot
AMMI2 polygon biplot
AMMI1 biplot
AMMI2 biplot
AMMI2 polygon biplot
n321.07718
n977.31036
Supplementary figure S2a

## Slide 3
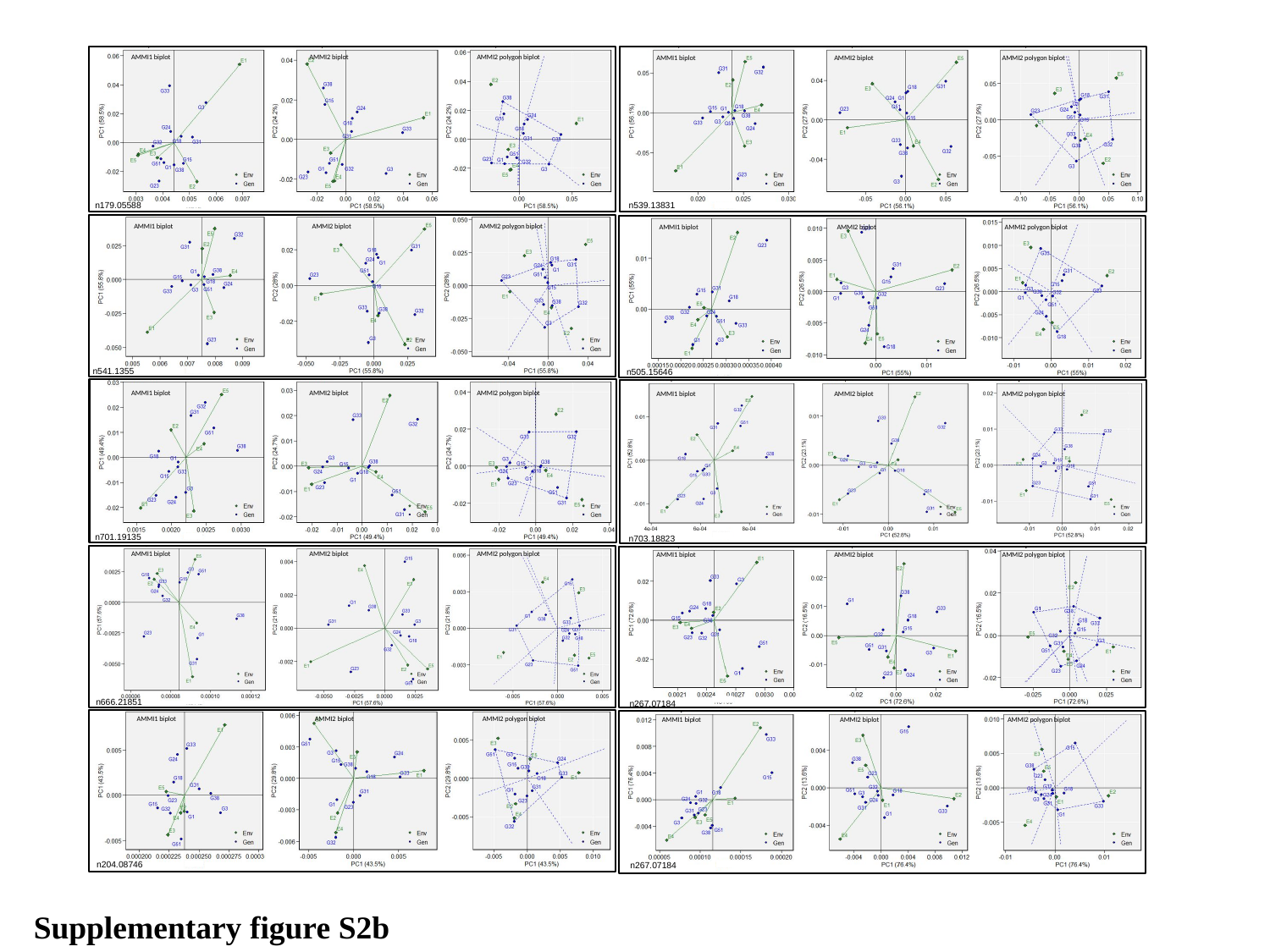

AMMI1 biplot
AMMI2 biplot
AMMI2 polygon biplot
AMMI1 biplot
AMMI2 biplot
AMMI2 polygon biplot
n539.13831
n179.05588
AMMI1 biplot
AMMI2 biplot
AMMI2 polygon biplot
AMMI1 biplot
AMMI2 biplot
AMMI2 polygon biplot
n541.1355
n505.15646
AMMI1 biplot
AMMI2 biplot
AMMI2 polygon biplot
AMMI1 biplot
AMMI2 biplot
AMMI2 polygon biplot
n701.19135
n703.18823
AMMI1 biplot
AMMI2 biplot
AMMI2 polygon biplot
AMMI1 biplot
AMMI2 biplot
AMMI2 polygon biplot
n666.21851
n267.07184
AMMI1 biplot
AMMI2 biplot
AMMI2 polygon biplot
AMMI1 biplot
AMMI2 biplot
AMMI2 polygon biplot
n204.08746
n267.07184
Supplementary figure S2b

## Slide 4
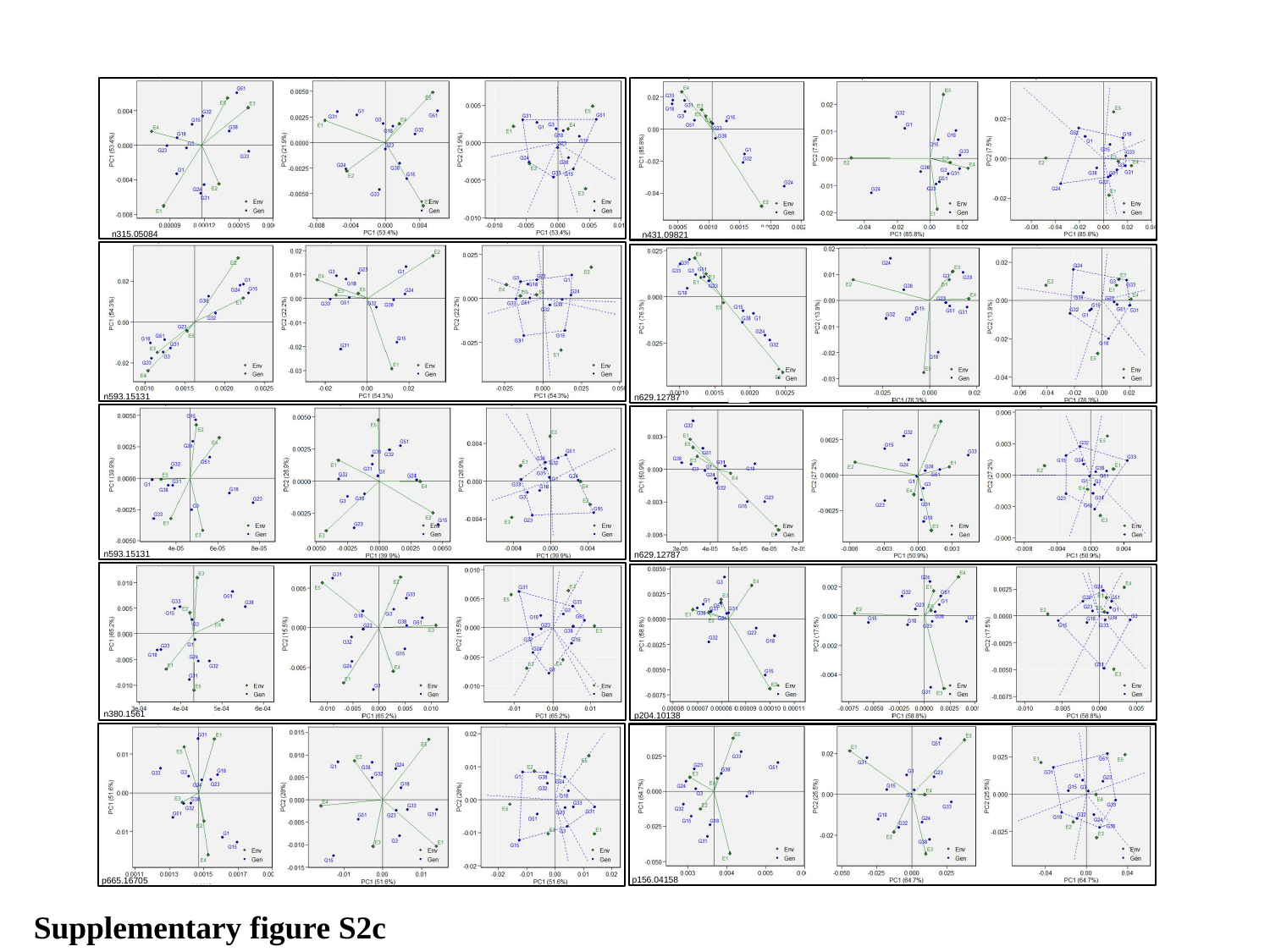

n315.05084
n431.09821
n593.15131
n629.12787
n593.15131
n629.12787
n380.1561
p204.10138
p156.04158
p665.16705
Supplementary figure S2c

## Slide 5
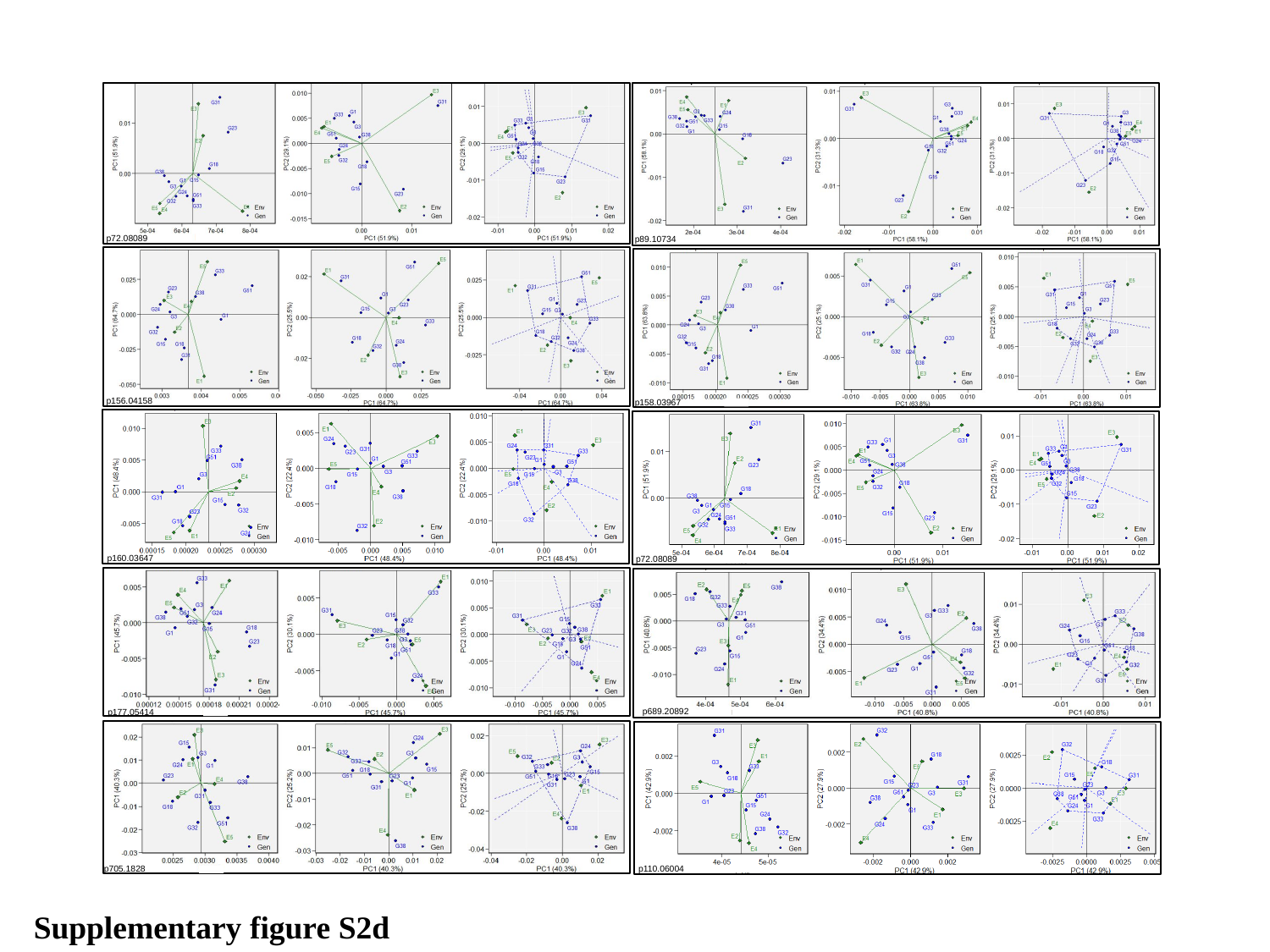

p72.08089
p89.10734
p156.04158
p158.03967
p160.03647
p72.08089
p689.20892
p177.05414
p110.06004
p705.1828
Supplementary figure S2d

## Slide 6
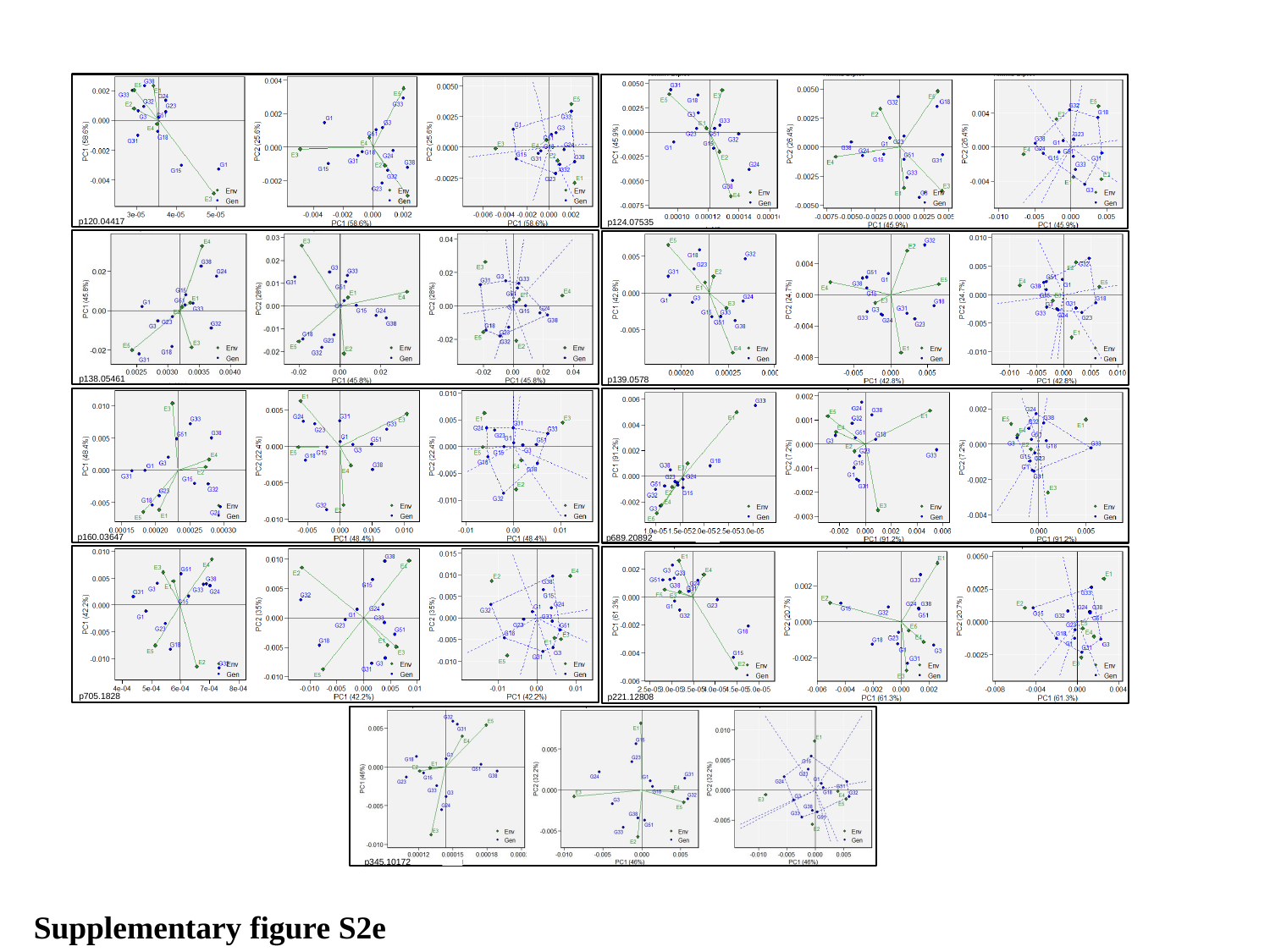

p120.04417
p124.07535
p138.05461
p139.0578
p160.03647
p689.20892
p705.1828
p221.12808
p345.10172
Supplementary figure S2e

## Slide 7
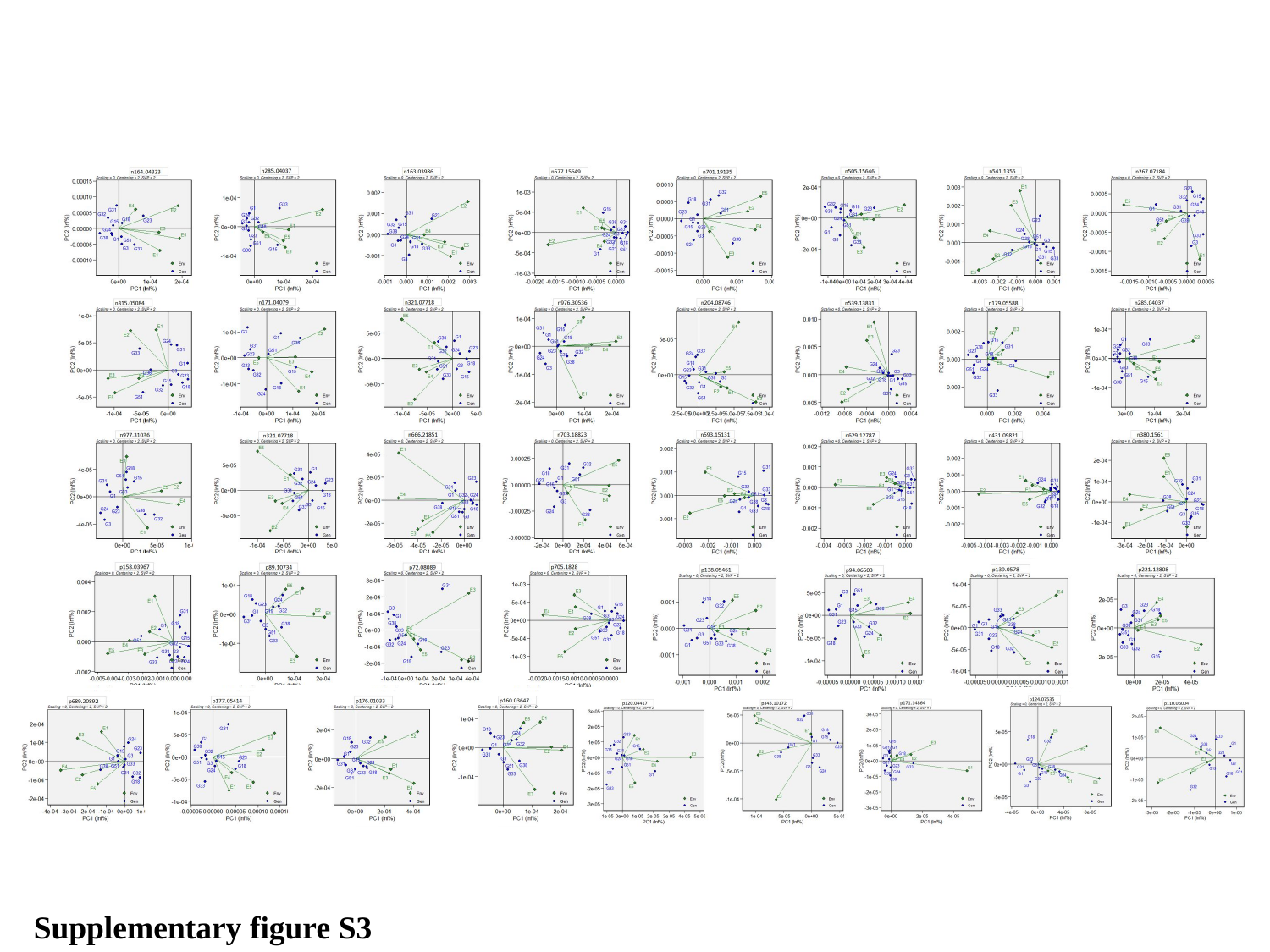

Supplementary figure S3

## Slide 8
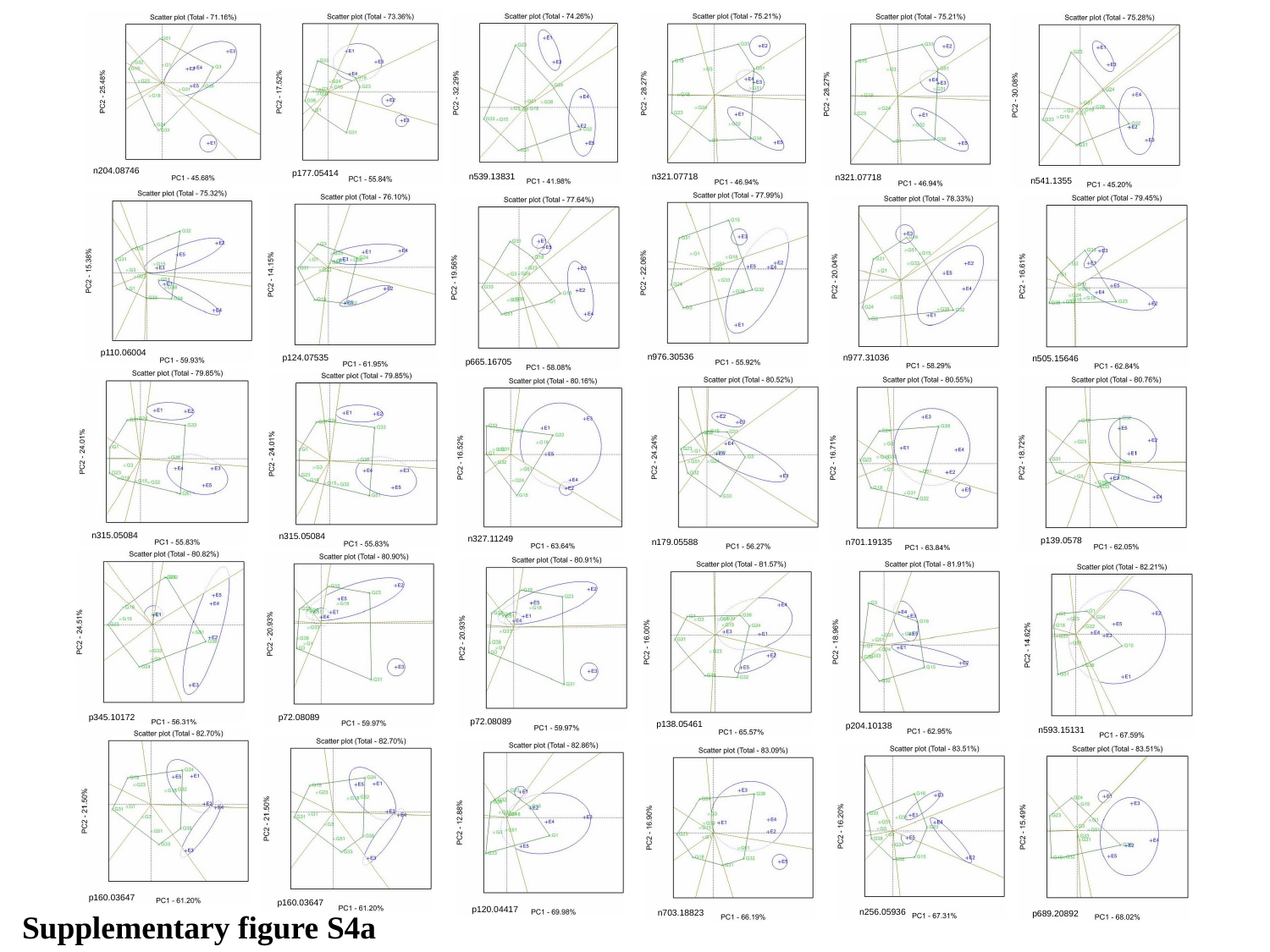

n204.08746
p177.05414
n539.13831
n321.07718
n321.07718
n541.1355
p110.06004
n976.30536
n977.31036
p124.07535
n505.15646
p665.16705
n315.05084
n315.05084
n327.11249
p139.0578
n179.05588
n701.19135
p345.10172
p72.08089
p72.08089
p138.05461
p204.10138
n593.15131
p160.03647
p160.03647
p120.04417
n256.05936
Supplementary figure S4a
n703.18823
p689.20892

## Slide 9
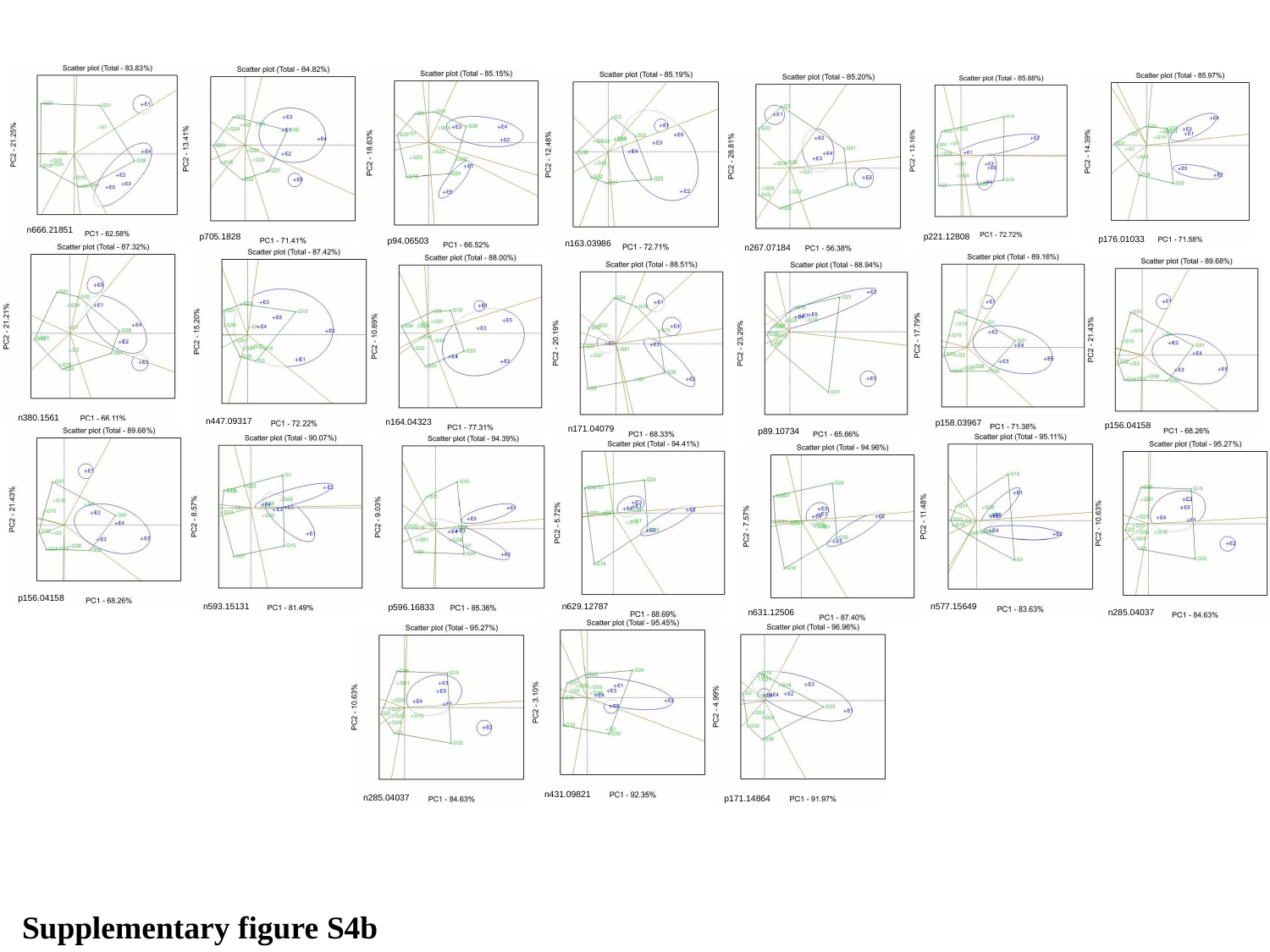

n666.21851
p705.1828
p221.12808
p176.01033
p94.06503
n163.03986
n267.07184
n380.1561
n447.09317
n164.04323
p158.03967
p156.04158
n171.04079
p89.10734
p156.04158
n593.15131
n577.15649
n629.12787
p596.16833
n631.12506
n285.04037
n431.09821
n285.04037
p171.14864
Supplementary figure S4b

## Slide 10
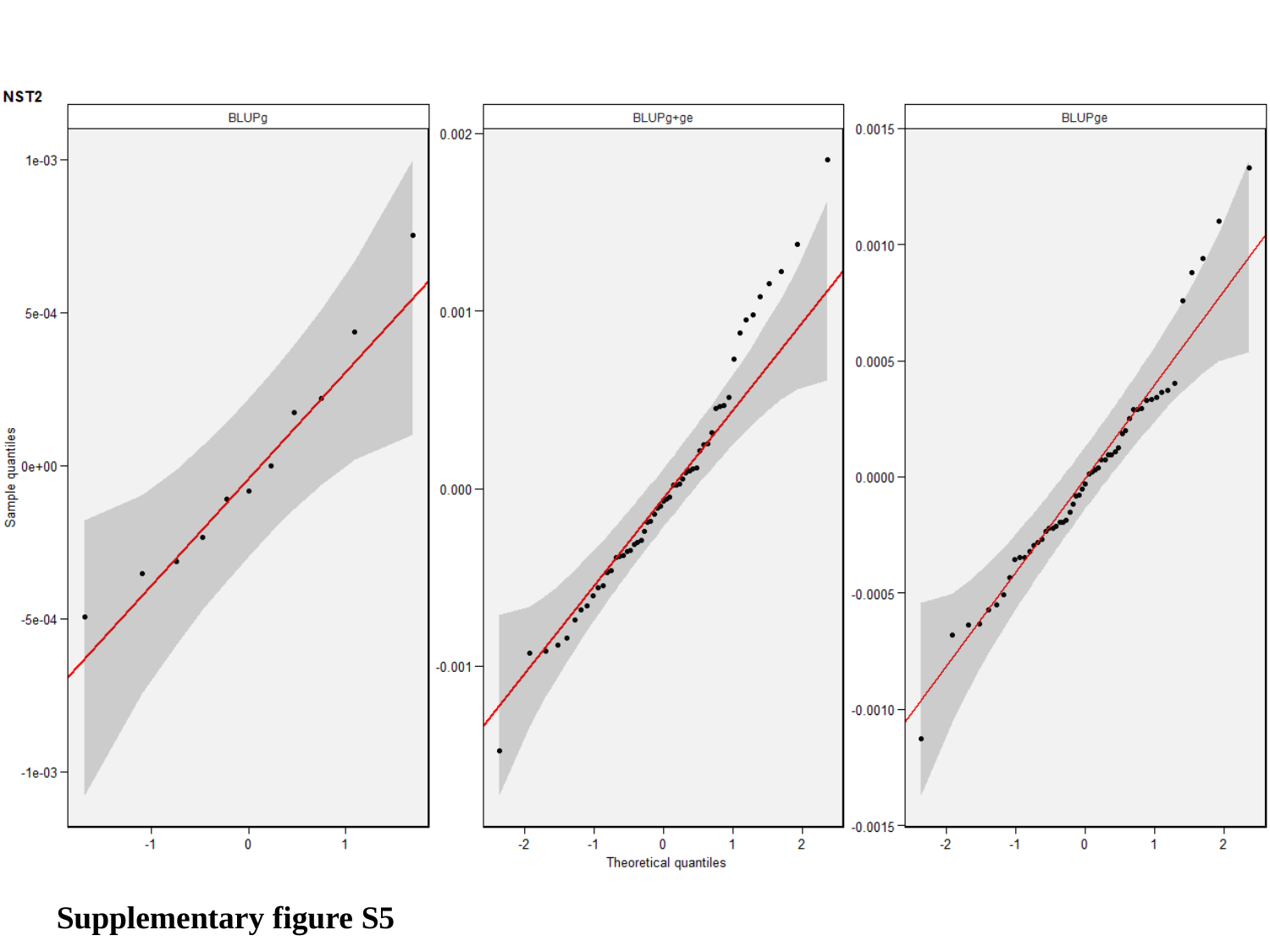

Supplementary figure S5
