# Supplementary material for: Genotype-by-Environment Interaction Analysis of Metabolites in Pearl Millet Genotypes with High Concentrations of Slowly Digestible and Resistant Starch in Their Grains
Source: Cells. 2022 Oct 2;11(19):3109. doi: 10.3390/cells11193109 (PMC9563316; doi:10.3390/cells11193109)
Supplement: Supplementary file 1 [file cells-11-03109-s001.zip › Table S4.pdf]

Supplementary Table S4 Combined analysis of variance for health benefiting metabolites significant at $p < 0.05$ in pearl millet.

m/z	Class	Genotype Variance	GenXLoc Variance	Environment Variance	Residual Variance	Grand Mean	LSD	CV
n164.04323	Antioxidants	1.7E-09	4.18E-10	1.21E-09	7.98E-09	0.000207	4.71E-05	43.21871
n285.04037	Antioxidants	1.2E-09	4.87E-10	1.92E-09	5.04E-09	0.000115	4.33E-05	61.63316
n163.03986	Antioxidants	3.73E-07	1.41E-07	1.89E-07	1.74E-06	0.002855	0.000723	46.17466
n577.15649	Antioxidants	3.81E-08	3.81E-08	6.81E-08	1.83E-07	0.000547	0.000291	78.27277
n315.05084	Antioxidants	5.51E-10	2.63E-10	1.28E-09	3.93E-09	0.000126	3.53E-05	49.69046
n171.04079	Antioxidants	1.11E-09	1.2E-09	1.74E-09	1.03E-08	0.000381	5.37E-05	26.60734
n321.07718	Antioxidants	2.97E-10	4.33E-10	1.18E-09	3.57E-09	0.000158	3.04E-05	37.71087
p596.16833	Antioxidants	5.17E-10	1.58E-10	8.64E-10	3.15E-09	4.63E-05	2.9E-05	121.2265
p156.04158	Antioxidants	4.86E-07	2.44E-07	1.82E-07	3.49E-06	0.003674	0.000914	50.81597
p665.16705	Antioxidants	1.32E-08	1.12E-08	5.37E-09	1.06E-07	0.00147	0.000168	22.1178
p204.10138	Antioxidants	7.9E-11	1.17E-10	1.53E-10	6.66E-10	8.3E-05	1.51E-05	31.0839
p89.10734	Antioxidants	3.32E-09	6.04E-09	2.68E-09	3.59E-08	0.000249	0.000102	76.04043
p72.08089	Antioxidants	5.17E-09	5.28E-09	3.85E-09	4.2E-08	0.000615	0.000134	33.33791
n976.30536	Fatty acid	1.52E-09	1.16E-09	3.45E-09	1.61E-08	0.000341	6.13E-05	37.26804
n977.31036	Fatty acid	1.98E-10	2.29E-10	3.93E-10	2.25E-09	0.000139	2.36E-05	34.12852
n321.07718	Fatty acid	2.97E-10	4.33E-10	1.18E-09	3.57E-09	0.000158	3.04E-05	37.71087
p158.03967	Fatty acid	1.8E-09	8.55E-10	5.99E-10	1.29E-08	0.000203	5.49E-05	55.92724
p156.04158	Fatty acid	4.86E-07	2.44E-07	1.82E-07	3.49E-06	0.003674	0.000914	50.81597
p160.03647	Fatty acid	1.48E-09	6.69E-10	1.62E-09	1.45E-08	0.000233	5.5E-05	51.76542
p72.08089	Fatty acid	5.17E-09	5.28E-09	3.85E-09	4.2E-08	0.000615	0.000134	33.33791
n666.21851	Starch	1.59E-10	6.41E-11	1.01E-10	1.18E-09	8.43E-05	1.65E-05	40.77659
n703.18823	Starch	9.03E-09	7.63E-09	1.67E-08	5.87E-08	0.000656	0.000142	36.92287
n701.19135	Starch	9.49E-08	8.47E-08	1.88E-07	6.27E-07	0.002212	0.000465	35.78216
n505.15646	Starch	2.61E-09	2.94E-09	1.44E-09	1.94E-08	0.000268	7.88E-05	51.99605
n541.1355	Starch	2.89E-07	3.85E-07	1.28E-06	4.16E-06	0.007515	0.000957	27.13685
n267.07184	Starch	3.98E-08	7.17E-08	8.51E-08	3.88E-07	0.002475	0.000355	25.14975
n204.08746	Starch	1.59E-10	2.01E-10	3.46E-10	2.83E-09	0.000238	2.28E-05	22.31806
n539.13831	Starch	2.53E-06	3.93E-06	1.24E-05	4.1E-05	0.023735	0.002937	26.99229
n179.05588	Starch	2.19E-07	4.05E-07	2.61E-06	2.88E-06	0.004418	0.000871	38.42367
n504.16653	Starch	6.99E-10	1E-09	2.49E-09	1.76E-08	0.000523	5.11E-05	25.38856
p705.1828	Starch	1.26E-07	3.74E-08	7.65E-08	6.59E-07	0.002935	0.000419	27.67346
p689.20892	Starch	4.08E-09	2.03E-09	1.22E-09	2.29E-08	0.000468	7.95E-05	32.36706
p177.05414	Starch	5.27E-10	3.89E-10	5.41E-10	7.13E-09	0.000174	3.67E-05	48.53046
n285.04037	Vitamins	1.2E-09	4.87E-10	1.92E-09	5.04E-09	0.000115	4.33E-05	61.63316
n593.15131	Vitamins	1.93E-07	9.91E-08	2.95E-07	8.85E-07	0.001623	0.000574	57.94342
n629.12787	Vitamins	1.86E-07	1.34E-07	2.4E-07	6.21E-07	0.001598	0.000578	49.32689
n431.09821	Vitamins	2.7E-07	2.19E-07	2.07E-07	7.05E-07	0.001059	0.000688	79.28266
n380.1561	Vitamins	3.61E-09	2.12E-09	2.01E-09	1.9E-08	0.000432	7.84E-05	31.93451
n327.11249	Vitamins	1.85E-10	8.16E-11	1.1E-10	1.41E-09	4.67E-05	1.81E-05	80.31226
n631.12506	Vitamins	1.7E-08	1.44E-08	2.2E-08	6.45E-08	0.00041	0.000183	61.9653

n256.05936	Vitamins	6.67E-11	4.98E-11	1.52E-10	5.48E-10	4.25E-05	1.23E-05	54.99522
n315.05084	Vitamins	5.51E-10	2.63E-10	1.28E-09	3.93E-09	0.000126	3.53E-05	49.69046
n447.09317	Vitamins	3.3E-09	3.08E-09	5.31E-09	2.94E-08	0.00027	9E-05	63.61719
p176.01033	Vitamins	8.06E-09	2.81E-09	5.88E-09	3.84E-08	0.000598	0.000108	32.78016
p160.03647	Vitamins	1.48E-09	6.69E-10	1.62E-09	1.45E-08	0.000233	5.5E-05	51.76542
p138.05461	Vitamins	1.36E-07	9.77E-08	1.76E-07	1.02E-06	0.003185	0.000539	31.72671
p94.06503	Vitamins	5.2E-10	3.31E-10	4.17E-10	4.45E-09	0.000146	3.29E-05	45.82165
p139.0578	Vitamins	8.35E-10	4.29E-10	7.5E-10	6.06E-09	0.000225	4.3E-05	34.57306
p221.12808	Vitamins	3.2E-11	3.75E-11	3.69E-11	1.77E-10	3.48E-05	8.73E-06	38.21265
p120.04417	Vitamins	2.9E-11	2.57E-11	6.16E-11	2.83E-10	3.56E-05	8.52E-06	47.29352
p345.10172	Vitamins	6.28E-10	3.79E-10	6.23E-10	4.77E-09	0.000147	3.85E-05	46.84449
p171.14864	Vitamins	2.46E-11	3.48E-11	3.97E-11	1.09E-10	1.56E-05	7.91E-06	66.71508
p124.07535	Vitamins	1.96E-10	1.84E-10	2.1E-10	2.08E-09	0.000121	2.23E-05	37.69499
p110.06004	Vitamins	1.7E-11	1.66E-11	2.37E-11	2.45E-10	4.41E-05	6.94E-06	35.49936
